# Scrofula; Its Nature, Its Causes, Its Prevalence, and the Principles of Treatment

**Published:** 1846-10

**Authors:** 


ART. III.—*Scrofula; its nature, its causes, its prevalence, and the principles of treatment.* By BENJAMIN PHILLIPS, F. R. S., *Assistant Surgeon to the Westminster Hospital.* Philadelphia, Lea and Blanchard. 1846. 8vo. pp. 350.

Of the diseases which may be said to constitute emphatically the *terra incognita* of Pathology, there are few which are more obscure than the affection known by the title of *Scrofula.* Few appellatives in Nosology are so vaguely used as this. It is not easy to designate with clearness the phenomena which it is intended to embrace, and it is applied practically with a corresponding degree of looseness, and diversity of signification. This fact is, in itself, sufficient evidence that our knowledge of the affection is lamentably deficient in completeness and precision; and if aught further were requisite to illustrate how little science has accomplished in elucidating its character, it would only be neccessary to adduce the various conflicting opinions which are entertained by different writers and practitioners respecting the pathological condition in which it is supposed to consist; and the agencies, internal and external, which are imagined to be instrumental in its production. Under these circumstances there is abundant occasion for fresh investigations, with the hope that new light may be shed on a subject which is as interesting and important as it is unfortunately obscure. Nor is it surprising that the very darkness and mystery which shroud this province of pathological inquiry should tempt the ardour of research, and invite some of the zealous spirits within the ranks of our profession to renewed explorations. Such appear to been the motives and aims of the author of the work whose title is prefixed to this article; and whatever value may be placed upon the results of his labors, it must be conceded that he has given proofs of a degree of patience and assiduity in the collection of data, and in the endeavor to develop from their investigation useful facts, for which he is deserving the highest praise, and which may be cited as worthy of imitation in the prosecution of this or any other branch of scientific inquiry.

Mr. Phillips informs us that he has for many years devoted particular attention to the study of scrofula, with reference to its nature, causes, and treatment. Being surgeon to an infirmary where peculiarly favorable opportunities for observing the disease in its various phases were presented, he was there led to a close examination of the phenomena which attend its progress, after it has become fully developed. But with a view to the facts appertaining to its etiology and prevalence, he was led, in the first place, to enter upon an extensive plan of inquiry, concerning the disease in different portions of his own country. For this he visited personally a large number of the national schools, parochial establishments, and charitable institutions of the metropolis; he inspected the weaving and factory districts, and procured returns from different portions of the country, embracing Scotland and Ireland. The data thus obtained were compared with the extensive mortuary tables, as analyzed and arranged by Mr. Farr, of England, and Mr. Wilde, of Dublin, and also, various hospital and dispensary reports. Not content, however, with limiting his researches to the British islands, in order to test the influences of different climates and habitudes, he prosecuted a very extensive system of inquiry into the facts connected with the origin and prevalence of the disease in various countries. He gathered information on these points from Russia, Austria, Prussia, Bavaria, Portugal, Holland, France, Belgium, Tyrol, Switzerland, portions of the United States of America, some districts in China, Egypt, Syria, and Greece. The author is therefore doubtless warranted in saying, that "the materials obtained and used are probably the most ample that have ever been collected and brought to bear on a single medical subject." The undertaking, from its magnitude, and the steady perseverance which it required, cannot fail to command admiration. We feel that we shall render to the readers of our journal an acceptable service, by presenting a digest of some of the more important facts and conclusions, which the author has derived from such a system of investigation, embracing, as it does, in extent of territory from which data were gathered, the greater portion of the habitable globe. We are compelled, however, in order to obviate disappointment, which might follow from too high expectations, to forewarn our readers that the positive results are not as brilliant as such extensive researches might lead them to anticipate. It must be confessed that science is but little, if at all, better qualified by these meritorious and able investigations, to explain the nature and the causes of scofula, and, it must be added, to indicate new and more effective methods of treatment. This, in effect, with great candour, the author himself confesses. The reader is not, therefore, to look for new and startling doctrines of the pathology and etiology of this disease. These remain as hidden and mysterious as ever. Still, we believe the work before us to be by no means devoid of important results. They are, however, in comparison with the discovery of new truth, of a negative character: that is to say, they consist chiefly in the disproval of a large proportion of opinions entertained, to a greater or less extent. These results, which for the sake of distinction we have called negative, are by no means less directly and positively useful than if they advanced our knowledge instead of correcting our errors. Science is equally on the pathway to truth, when she recedes from false doctrines, as when she progresses by means of newly developed fact; and the labors by which the former is promoted are as valuable as is the genius which accomplishes the latter.

With these remarks by way of introduction, we proceed to give a condensed summary of the conclusions to which our author arrives, by an analysis of the data which he has accumulated, submitting incidentally such reflections and critical observations of our own, as may be compatible with a due regard to the limits within which we must confine ourselves.

Mr. Phillips devotes a chapter to a rehearsal of the ancient ideas with regard to scrofula. These present considerable diversity, but they have this in common, viz. they are all based on the crudest assumptions. None have resulted from a "scientific investigation of phenomena, or a philosophical deduction from reasonably established facts." They may all, however, be arranged into two classes. In one class the disease was regarded as a local affection, in the other it was deemed constitutional, the local affections being considered in the light of symptoms, or manifestations of the general disorder. Present opinions may be arranged in the same classes, although they are few in number who now consider the disease primarily local. The major part of the profession, doubtless, view it as a constitutional disease; but whether the pathological condition essential to its existence be in the fluids or solids, is a question upon which medical writers and practitioners are more equally divided. On this point some remarks presently. The author's "own ideas of the nature of scrofula," which occupy a distinct chapter, are, that it "is a disease of the constitution, and that it is most clearly manifested by certain external signs, of which swelling of the subcutaneous lymphatic ganglia is the most conclusive." The latter clause of this quotation may serve as introductory to a distinctive feature of the author's views, and as the subject is intimately involved in all his researches, and is dwelt upon at considerable length in the work, it will be proper to bestow upon it some attention. It is well known that the external characters denoting scrofulous disease possess great variety, and there are few, if any, which are so uniformly associated with it as to constitute unequivocal indications of its existence. But for many reasons it is desirable to fix upon some criterion, and especially in the collection of statistics this is absolutely necessary for the sake of uniformity, and to avoid the imputation of incorrectness in the data, owing to the great looseness with which the term scrofula is habitually applied by practitioners. Mr. Phillips adopts as the distinctive mark of the disease, *the presence of the peculiar deposit known as the scrofulous matter in one or more of the subcutaneous lymphatic glands.* He would limit the application of the term scrofula to cases in which the presence of this deposit is demonstrable, that condition of the economy which precedes this deposit being, not scrofula, but a certain diathesis or disposition, which, after a greater or less duration, may terminate in scrofula, or, on the other hand, may disappear without that product being developed in the lymphatic ganglia which is necessary to constitute the disease in question. Granting that as a characteristic sign for the purpose of securing correctness in statistical returns, no other could have been selected which would have been better adapted to the object, we are unable, beyond this, to perceive any propriety, or practical benefit, in the principle or doctrine just referred to. As the author states, it does not follow from the fact that the subcutaneous lymphatic glands become tumid, that they are enlarged from scrofulous disease. A variety of other causes may induce this effect. Even in scrofulous cases, an indefinite period may elapse before the nature of the deposit is determinable ; and, in the mean while, are we only to entertain a suspicion that the disease is scrofula? According to the views of the author himself, the disease is constitutional, not local ; the scrofulous deposit is only a product dependent on ulterior changes. Assuredly, then, if any thing is to be hoped for from therapeutical management, it is important to arrive, if possible, at a diagnosis without waiting for the elaboration and discharge of this specific matter. But is the presence of tumid subcutaneous glands even essential to the existence of scrofula? We suspect there are few physicians who will answer this query in the affirmative. Every practitioner is in the habit of meeting with cases in which he is authorised, by combined circumstances, to set down the disease as scrofula, in the absence of this manifestation. In other words, the affection of the subcutaneous ganglia is but one of the various expressions of the constitutional disease. It is the most frequent, and, perhaps, the least equivocal, of any of the local expressions taken singly, but it is, after all, only a contingent symptom, not essential to the integrity of the disease. These are the views which, if we do not err, are generally entertained, and which are most consistent with the knowledge we possess on the subject. At the same time, we are free to confess that we are not prepared to point out any other indication, or even any collection of symptoms, which would have answered better as an uniform criterion for cases to be accumulated from various sources for statistical comparison. Our criticisms relate, of course, to the exclusiveness of considering this sign the only proof of the disease for ordinary, practical purposes, and the inconsistency of detaching from the nosological space which the term scrofula occupies, those cases wherein other diagnostic marks are present, this manifestation being absent.

Adopting, as the author does, the presence of the scrofulous matter in the lymphatic ganglia as the only certain diagnostic, he does not, of course, deem it necessary for purposes of diagnosis to devote much attention to the other external characters which belong to the disease. On this point there is confessedly great deficiency of precise knowledge; yet, if the criticism which we have just submitted be just, we are sometimes required to form a diagnosis without the aid of glandular swellings, and we are called upon to diagnosticate from other external characters in cases in which glandular enlargements exist, but in which we are unable to satisfy ourselves that they contain the true scrofulous deposit. Hence, the study of *all* the circumstances which may bear directly or indirectly on diagnosis, is a matter of much importance. We regret that the author did not permit this to enter into his researches to a greater extent. The cursory manner in which the subject is passed over, is a serious defect in the work. He gives a brief summary of the more prominent circumstances which may serve as indications, but there is nothing sufficiently novel or important to warrant a quotation. In leaving the subject of diagnosis, we would simply remark, that with respect to this, as to several other forms of disease in which the essential pathological condition eludes investigation, we are to be guided, not by any single sign, but by a catenation of symptoms. Individually, each may be, and doubtless often is, fallacious, because no one is an invariable concomitant of the disease; and were we to trust to any one singly, we should often be deceived. But a collection of phenomena, each having more or less significancy individually, will, in the majority of instances, with the exercise of proper caution and judgment, enable the practitioner to arrive at a just conclusion.

We proceed to enumerate the captions of the chapters successively, giving under each the gist of the author's investigations and inferences.

*The Scrofulous deposit — its Physical characters.* — "To the naked eye, this matter is presented in the form of an amorphous, grayish, buffish, or yellowish mass, irregularly granular, and not unlike moist old cheese." Under the microscope it has a granular appearance; beyond this the observations of microscopical observers lack precision and agreement.

*Chemical Characters.* — "It is mainly composed of albumen, fat or oil globules, and certain alkaline salts." With regard to physical and chemical characters, the author's *resume* is as follows:

"So far as regards the physical and chemical properties of scrofulous deposits, which although varying with the particular structure of the organ, yet in so far as concerns a simple inspection, present considerable uniformity, and they do not yield any uniformly differential character when subjected to minute analysis, either by the microscope or by chemistry."

*State of the organ in which the product is about to be deposited.* — "Commonly, if not always, glandular structures undergo considerable change before scrofulous matter is deposited in them. They acquire a considerable increase in volume, in density, and in vascularity. The bulk may be ten or twenty times what is natural to them, the density may equal or exceed that of veal, and the change of color from increased vascularity is very remarkable."

He concludes from examinations of these ganglia, that an inflammatory condition precedes the deposition of scrofulous matter, but whether this condition is independent of the circulation of blood within them which has undergone change, he declares himself unable to decide.

*Sources from whence scrofulous matter is derived.* — Here arises a highly interesting and important inquiry: Is the scrofulous matter already present in the blood, and simply excreted into the gland, or is it, strictly speaking, a product of glandular action, the elements only being derived from the circulating fluid? This question would be, perhaps, considered satisfactorily settled, had the matter been demonstrated to exist in the blood; although, in that case, the objection might be urged, that it was primarily a product of the ganglia, which afterward gained entrance into the vessels. No one, however, but Lugol, has claimed to have observed its presence in the blood. Until confirmed by the observations of others, caution dictates a distrust of the fact, not doubting the good faith of Lugol in his assertions. He may easily have been deceived. The question cannot be regarded as susceptible, at present, of a satisfactory answer. It seems most rational, however, for a variety of reasons, to believe that depravation of the blood necessarily precedes the deposit, and, in fact, constitutes the fundamental pathological condition upon which the disease depends. This may be true without the necessity of the scrofulous matter being actually formed within the blood vessels. Certain changes may have taken place which determine the formation of the scrofulous matter, the agency of the glands being still essential to its production. Mr Phillips inclines to the opinion that the blood is affected prior to the ganglia. He has bestowed particular examination upon the blood in a considerable number of cases, with the following results:

"I have examined the blood in sixty-seven instances of scrofula, and although I have almost always observed a considerable deviation from the condition of healthy blood, the changes have not presented sufficient uniformity to induce me to regard any particular condition as specially characteristic of scrofula; the changes are such as seem to belong to a tolerably extensive group of affections, all, it is true, being connected with disordered nutrition and debility.

"In almost every case, the coagulum was relatively small, the serous menstruum large, the clot was usually very soft, almost diffluent; in a few instances only it was tolerably firm. In almost all cases, the proportion of globules was considerably under the healthy standard. The fibrin had not usually undergone much change; in a few cases it exceeded, in many more it was below the healthy standard. In most instances there was a considerable increase in the proportion of albumen; in almost every instance the proportion of salts was found to exceed the healthy standard; in some instances it was nearly doubled. At one time I thought I had clearly made out, that the proportion of chloride of sodium was deficient, and certainly in many cases it was so ; but in many other cases that deficiency was not evident."

His general conclusion is, that although it may be fairly assumed that the blood is changed before the deposit is made, yet, we know of no particular condition which clearly marks the existence of scrofula.

*Are Pulmonary Tubercle, or Phthisis, and Scrofula in their nature identical?*—The opinion that scrofula and tuberculosis are very closely allied, has been maintained by many able writers, and is very generally entertained by practitioners. This opinion has doubtless been, in a great measure, strengthened by the fact, that the scrofulous and tuberculous matter present very similar appearances. They both, agreeably to the common ideas on the subject, involve a constitutional taint or cachexy, from which the local affections originate, and it is customary to attribute each to similar causes acting on the organism. Our author, however, considers this community of nature, causes, &c., by no means established, but, on the other hand, opposed by several cogent arguments.

As regards general physical characters determinable by the unaided senses, the appearances revealed by the microscope, and chemical differences, no well-marked points of distinction can be satisfactorily made out. In short, as to the products themselves the author finds but little to sustain his opinion. He thinks there is a difference in the condition of the part in which the deposit is made. In scrofulous glands the organ becomes enlarged, more vascular, and its consistence is almost flesh-like—in short, it is the seat of an inflammation. On the other hand, we have not evidence that in all cases inflammatory action precedes the deposit of tuberculous matter in the lungs. It does not appear to us that he establishes very clearly this point of distinction. With reference to vascularity of the products as a source of difference, there is some discrepency in the observations of different persons. The probability is, that in neither case is the matter itself capable of being injected. In other circumstances however, than those relating to the specific products of the disease, some striking points of difference are adduced, In the first place, it is satisfactorily established by mortuary tables, what is familiar to common observation, that scrofula, in cases in which it proves destructive to life, occurs much more frequently before than after puberty, while the ravages of Phthisis are much greater after than before puberty. By reference to registration of deaths, also, the curious fact is disclosed that while Phthisis falls with greater severity upon females, the liability to scrofula is the reverse — males being afflicted by it in greater number. A striking difference in their relative frequency is, also, observable. The mortuary tables of Great Britain show that where there is a large mortality from Consumption there is a small mortality from scrofula, and *vice versa*. Assuming that the figures are to be received as true expressions of the facts, it is difficult to reconcile these results with the supposition that the causes of the two affections are the same. Again, the proportion of those with Phthisis whose bodies exhibit traces of previous scrofulous affections is very small. On this, as on the other points of statistics, Mr. Phillips gives the results of extensive researches. Finally we give his general inferences in his own words.

"I apprehend it has now been shown, by abundant evidence, that with the exception of the deposit itself, which, whether found in the lungs, or in a cervical gland, whether examined by the naked eye, by the microscope, or by chemical analysis, is very similar, the circumstances attendant upon the development of Scrofula and Phthisis are widely different. In Scrofula the gland undergoes considerable change, inflammatory in its nature, before the matter is deposited in it; in the lung we commonly find the tissue around a recent simple tubercular deposit unchanged by inflammation. We find, further, that in districts where the causes of Phthisis act with most intensity, those of Scrofula fall lightest; that the age when the ravages of Scrofula are most keenly felt is precisely that when the visitation of Phthisis is least to be apprehended; that the sex which suffers most severely from one of those diseases is least affected by the other. And beyond all this, there is the fact that among the numerous victims of Phthisis, at least eighteen out of every twenty exhibited no marks of having suffered from Scrofula. It seems to me, therefore, that these facts constitute so clearly marked a difference between the two affections, that it will be most convenient, most conducive to scientific correctness, to consider them as affections possessing a certain general similarity of character, but no identity. It may be that they belong to the same family, as do Pleurisy and Pneumonia; but every one deems it desirable to make as clear a demarcation as possible between those diseases. I say the same of tubercular disease generally and Scrofula, between which the points of resemblance are strong, in so far as concerns the deposit; but in all else they are weak."

*Prevalence of Scrofula in Great Britain, and in other Countries*—In order to ascertain the prevalence of the disease in his own country, Mr. Phillips examined, either himself, or by the aid of others, a large number of children in Schools, Union Houses, Factories, and elsewhere ; he availed himself of Hospital and Dispensary returns ; he procured from the Army Medical Board returns of the prevalence of marks of Scrofula among recruits ; also returns respecting the inmates of prisons ; and he consulted the reports of the Registrar General on the subject. The general results are as follows :

"The proportion of children, between the ages of five and sixteen, who present marks of Scrofula, evident upon a simple inspection, amount to as near as may be, but rather under 3 1-2 per cent. The marks to which I refer are glands sufficiently enlarged to be obvious upon simple inspection in the absence of any evident local cause of irritation; scars resulting from their suppuration, or diseased bones and joints, evidently scrofulous in their character. But that proportion does not represent the actual prevalence in the whole population, for among adults similar marks are not found to exist in a greater proportion than 1 1-2 per cent. Taking the gross population of the country, such marks are not observable in quite 2 1-4 per cent. of the people."

*Among Hospital and Dispensary patients* the registers of physicians and surgeons consulted, show the following numbers : "Of 1000 cases on the physicians' books, 19 were registered scrofula; of 2000 cases on the surgeons' books, 112 were cases of scrofula." This is nearly 4 per cent. of the total admissions.

*Among Recruits*, the returns show that of 95,586 examined, all having the requisite height, 800, or 1 in 119, were rejected for marks of scrofula.

*Among Convicts.* In the year 1840, of 1952 prisoners received at the Penitentiary, 14 presented at the time of their admission scrofulous diseases of the external glands. Of 660 boys at the Parkhurst Prison, 95 presented enlarged glands.

*Registered Mortality.* This shows 8 per 100,000 on the gross population of England and Wales.

The author justly remarks that the concurrence in the results collected from these several sources, constitutes a strong ground for supposing that the data collected actually represent the prevalence of the disease. And he further deduces that "unless scrofula degenerates into some other disease, its prevalence as well as its influence in the destruction of human life in Great Britian is not very formidable." The prevalence of the disease in different districts of England and Ireland is by no means equal. Among poor children the variation is from 11 to 72 per cent. An unexpected result of the comparison of statistics is, that in manufacturing districts, the per centage of deaths registered from scrofula, is considerably less than in agricultural districts.

As regards the prevalence in other countries, he submits an analysis of returns from various countries. We will quote the results of several. At Lisbon, on examination of 800 children, the proportion affected, was, boys, 50 per cent. girls, 10 per cent. At Amsterdam, 495 children examined, presented 42 per cent. with the ordinary marks of the disease. At Vienna, out of 412 children, 11 per cent. were treated for scrofula in 1841. At Berlin, of 353 children, 53 per cent. At St. Petersburgh, 840 children, 41 per cent. Moscow, children examined, 15,515, 9 per cent. At Paris, by reference to mortuary tables, it is found that taking a series of years, the deaths from scrofula amount to 1 in every 3221 of the population. In Geneva, to 1 in every 2790. In London, they amount to but 1 in 9,000.

The returns from America presented striking variation. They were furnished from the house of Industry, at Boston; and from an examination of school children at Philadelphia, made under the supervision of Dr. S. Jackson. The former included 146 children, of whom 70 per cent. exhibited marks of scrofula. The latter shows a total of 2998 children examined, of whom only 13 are returned as scrofulous! This is certainly a striking contrast, and if we were to deduce any inferences, we should conclude either that the house of Industry embraces an unusual proportion of scrofulous children, or that the disease is vastly more prevalent at Boston than at Philadelphia. But the truth is, to draw any satisfactory deductions, the data should be much more extensive. Examinations should embrace a larger number, comprehending different localities, a variety of external conditions, and a succession of years. The returns from this country are entirely insufficient to determine the prevalence of the disease. It is fair to presume, also, that the data are more or less imperfect, as furnished from other countries. The author has taken great pains to procure extensive and correct information, but from the difficulties necessarily inherent in the undertaking, the results are only to be considered as partial approximations to the truth, and are to be received with great allowances for errors and incompleteness.

The general conclusion to which he arrives from a comparison of figures is, that "the notion that scrofula is eminently an English disease, is incorrect, and, that there is no country, so far, at least, as our information extends, in which the people are more free from the disease than in England and Wales."

*Is the occurrence of Scrofula proportionally more frequent than formerly?*—The data for comparisons to determine this point are too few and imperfect to give any reliable results. The bills of mortality extending backward nearly two centuries, which, however, comprised but a portion of the city of London, and are doubtless very inaccurate, were consulted by Mr. Phillips, and also the register of persons who received the Royal touch for the disease, from May 1667, to May 1664. The following is his conclusion:

"Tried, then, by such tests as I have been enabled to apply, which though not strictly accurate, are the best we possess, and which, when used with caution, constitute a fair body of evidence on the point, the conclusion seems a fair one that Scrofula is much less prevalent in the present day than it was in the seventeenth and eighteenth centuries."

Having occupied as much space as we can well devote to this subject in the present number, we will defer our analysis of the remainder of the work for the following month. The topics which remain are the causes of scrofula and the principles of treatment.

